# Pulse-Wave Analysis of Optic Nerve Head Circulation Is Significantly Correlated with Kidney Function in Patients with and without Chronic Kidney Disease

**DOI:** 10.1155/2014/291687

**Published:** 2014-01-02

**Authors:** Tomoaki Shiba, Mao Takahashi, Takatoshi Maeno

**Affiliations:** ^1^Department of Ophthalmology, Toho University Sakura Medical Center, 564-1 Shimoshizu, Sakura, Chiba 285-8741, Japan; ^2^Cardiovascular Center, Toho University Sakura Medical Center, 564-1 Shimoshizu, Sakura, Chiba 285-8741, Japan

## Abstract

*Aim.* To determine whether there is a significant correlation between the optic nerve head (ONH) circulation determined by laser speckle flowgraphy (LSFG) and kidney function. *Materials*. Seventy-one subjects were investigated. The estimated glomerular filtration rate (GFR) and serum creatinine, cystatin C, and urinary albumin excretion were measured. The ONH circulation was determined by an analysis of the pulse wave of LSFG, and this parameter was named blowout time (BOT). Chronic kidney disease (CKD) was defined to be present when the estimated GFR was <60 mL/min per 1.73 m^2^. Pearson's correlation coefficients were used to determine the relationship between the BOT and the kidney function. We also examined whether there were significant differences in all parameters in patients with and without CKD. *Results*. BOT was significantly correlated with the level of creatinine (*r* = −0.24, *P* = 0.04), the estimated GFR (*r* = 0.42, *P* = 0.0003), cystatin C (*r* = −0.29, *P* = 0.01), and urinary albumin excretion (*r* = −0.29, *P* = 0.01). The BOT level in subjects with CKD was significantly lower than that in subjects without CKD (*P* = 0.002). *Conclusion*. BOT in ONH by LSFG can detect the organ damage such as kidney dysfunction, CKD.

## 1. Introduction

Chronic kidney disease (CKD) is associated with high cardiovascular mortality [1–3]. More than 45% of predialysis CKD patients die with cardiovascular disease before reaching the end stage of kidney disease [4]. CKD has been defined as a creatinine-based estimated glomerular filtration rate (GFR) that is <60 mL/min per 1.73 m^2^ [5]. This represents a loss of more than one-half of normal renal function. An estimated GFR of <60 mL/min per 1.73 m^2^ has been strongly correlated with cardiovascular risk and death [3].

Other factors reflecting kidney function are the levels of serum cystatin C and urinary albumin excretion. Evaluations of serum cystatin C and serum creatinine were reported to predict cardiovascular events in elderly persons without chronic kidney disease [6]. In addition, urinary albumin excretion is a predictor of all causes of mortality in the general population [7]. However, the relationship between these kidney function parameters and ocular circulation remains unclear. Therefore, it was suggested that evaluations of the relationships between ocular circulation and kidney function are important, because kidney and eye reflect the peripheral circulation in both.

Laser speckle flowgraphy (LSFG; Kyushu Institute of Technology, Fukuoka, Japan) is a method of determining ocular blood flow and is based on the changes in the speckle pattern of laser light reflected from the fundus of the eye [8, 9]. LSFG is dependent on the movement of erythrocytes in the retina and choroid [10], and the mean blur rate (MBR) is a measure of the relative velocity of the erythrocytes. Changes in the MBR have a pulse-wave pattern that is synchronized with the cardiac cycle.

The purpose of this study was to determine whether there is a significant correlation between the optic nerve head circulation determined by LSFG and the estimated GFR, serum creatinine, serum cystatin C, and urinary albumin excretion. In addition, we examined whether there are significant differences in the optic nerve head circulation in patients with and without CKD.

## 2. Materials and Method

One hundred and thirty-six consecutive subjects who visited the Vascular Function Section of the Department of Cardiovascular Center of Toho University Sakura Medical Center between April 1, 2007, and October 1, 2010, were studied. The exclusion criteria were subjects who had diabetes mellitus, history of cardiovascular disease and heart failure, presence of cerebrovascular disease, arrhythmias (atrial fibrillation), history of ophthalmic surgery, cataract which affects the corrected visual acuity, glaucoma, and vitreous and retinal diseases. Subjects who had systemic hypertension were included. In the end, 71 patients whose mean ± standard deviation (SD) age was 62.3 ± 10.5 years with a range from 39 to 86 years met the study criteria. There were 48 men and 23 women. And all subjects had a corrected visual acuity exceeding 20/20.

The Institutional Review Board of Toho University Sakura Medical Center approved the protocol of this study, and we began the research after all subjects received information on the purpose and possible side effects of the research protocol and signed an informed consent form. The procedures used conformed to the tenets of the Declaration of Helsinki.

The LSFG was measured after the patients rested for 10 minutes in a quiet and air-conditioned room maintained at 24°C. Measurements were made between 15:00 to 17:00 hours before a meal. All subjects abstained from alcohol, smoking, and caffeine for at least 12 hour prior to the measurements.

### 2.1. Measurement of Kidney Function

Kidney function was assessed by determining the level of serum cystatin C and creatinine. The creatinine-based modified diet of renal disease of the estimated GFR was calculated using the equation for Japanese subjects recommended by the Japanese Society of Nephrology: estimated GFR (mL/min/1.73 m^2^) = 194 × SCr^−1.094^ × age^−0.287^ (×0.739 if female) [11, 12], and urinary albumin excretion. Fasting morning blood samples were collected and stored at −70°C until needed for the appropriate assays. The serum cystatin C was determined by fluorescent enzyme immunoassay (ST AIA-PACK cystatin C; TOSOH Corporation, Tokyo, Japan). The serum creatinine was determined by an enzymatic method. CKD has been defined to be present when the estimated GFR is <60 mL/min per 1.73 m^2^ [5]. The urinary albumin excretion was determined in an early morning urine sample.

### 2.2. Systemic Parameters

The following systemic parameters were measured: age, systolic blood pressure (SBP, mmHg), diastolic blood pressure (DBP, mmHg), pulse pressure (mmHg), heart rate (beats/min, bpm), and body mass index (BMI, kg/m^2^). The mean arterial blood pressure (MAP, mmHg) was determined by the formula; MAP = DBP + (SBP-DBP)/3.

### 2.3. Pulse-Wave Analysis of Optic Nerve Head Circulation Using LSFG

The determination of the LSFG from these images has been described in detail [8, 9, 13]. Briefly, the LSFG is obtained from a 21° section centered on the optic disc. This total of observation field is made up of 750 (width) ×  360 (height) pixels. LSFG uses the mean blur rate (MBR) as an indicator of the relative velocity of the erythrocytes. The MBR is determined by the following formula: MBR = 2 × (normalized  blur  rate)^2^.

**Figure 1:**
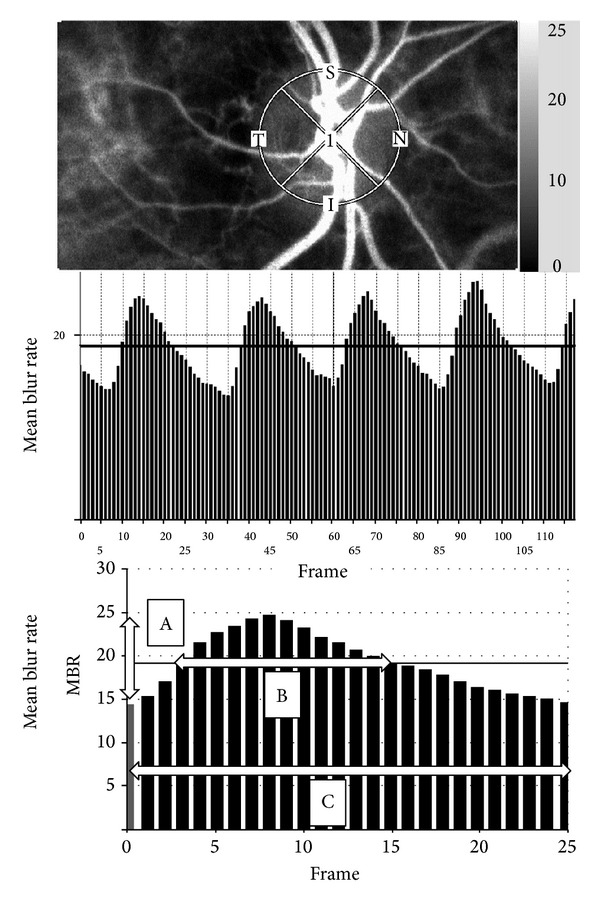
Method of determining the pulse-wave velocity in the optic nerve head circulation by laser speckle flowgraphy (LSFG). Upper panel: a gray scale photograph of the fundus of the eye. the circle designates the area that was measured in the optic disc area. Middle panel: pulse waves showing changes in the MBR which is tuned to cardiac cycle for 4 seconds. The total number of frames is 118. Lower panel: normalization of one pulse. A = maximum MBR–minimum MBR. B = number of frames spent at one-half the value of A. C = number of frames spent at normalized one pulse.

The normalized blur rate is calculated from the speckle pattern generated by reflected lights from the moving erythrocyte illuminated by a 830 nm wavelength diode laser beams [8, 10]. Initially, 118 MBR images (118 frames) were recorded in 4 seconds from the optic nerve head area. Next, a gray scale map of the still images was made by averaging the MBR images (Figure 1, upper panel). On the analysis screen, the pulse wave of the changing MBR which corresponded to each cardiac cycle was obtained (Figure 1, middle panel). Finally, analysis of the screen which is normalized to one pulse is displayed (Figure 1, lower panel), and the analysis of the pulse wave in the optic nerve head circulation is made on this screen.

The maximum MBR-minimum MBR waves are labeled as “A” and are shown in the lower panel of Figure 1. The number of frames spent at one-half of the value of A is designated as “B,” and the number of frames spent for one cardiac cycle is labeled “C.” The time analysis of pulse wave in the optic nerve head circulation was determined by the formula blowout time = 100 × (B)/(C) [14, 15].

This parameter is named the blowout time (BOT), and we measured the BOT of the optic nerve head area three times and used the average for the statistical analyses. All subjects were measured in seated position and pupils were dilated with 0.5% tropicamide eye drops. And only the data from the right eye were used for the analysis.

### 2.4. Measurements of Other Ocular Parameters

The following ocular parameters were also studied: the intraocular pressure (IOP mmHg) measured by applanation tonometry and ocular perfusion pressure (OPP mmHg) which was defined as OPP = (2/3MAP) − IOP.

### 2.5. Statistical Analyses

Data are presented as the means ± standard deviations (SDs) for the continuous variables. Pearson's correlation coefficients were used to determine the relationship between the BOT, kidney function, and other systemic and ocular parameters. In addition, we examined whether there were significantly differences in all parameters in patients with and without CKD. Statistical analyses were conducted using the Yates 2 × 2 chi-square test, unpaired *t*-test, and Mann-Whitney *U* test. A *P* value <0.05 was considered to be statistically significant. The Stat View version 5.0 program (SAS Institute Inc., Cary, NC, USA) was used for the statistical analyses.

## 3. Results

The results of the systemic and ocular measurements are shown in Table 1. Forty of the 71 (56.3%) subjects had hypertension. The mean ± standard deviation (SD) of the BOT was 47.8 ± 3.1 with a range from 41.6 to 53.9 for all of the subjects (Table 2).

**Figure 2:**
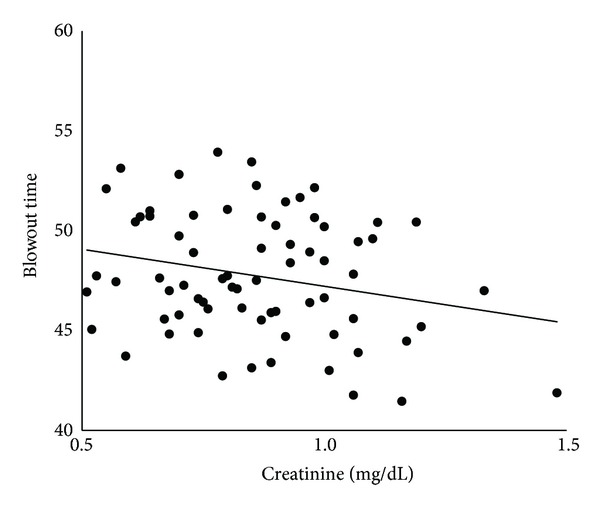
Correlation between blowout time and serum creatinine in all subjects.

**Figure 3:**
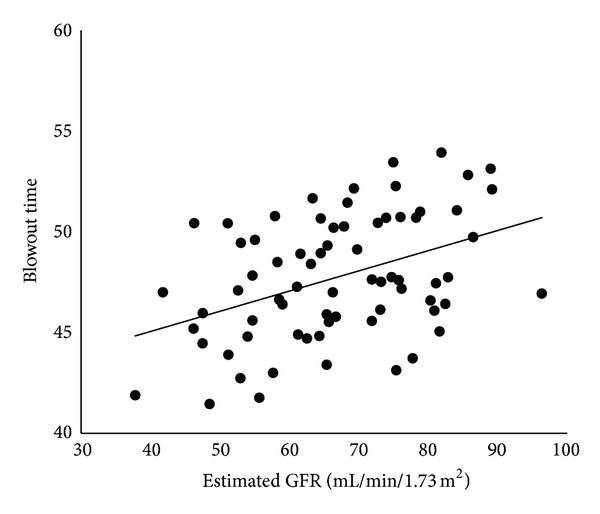
Correlation between blowout time and estimated glomerular filtration rate (GFR) in all subjects.

**Figure 4:**
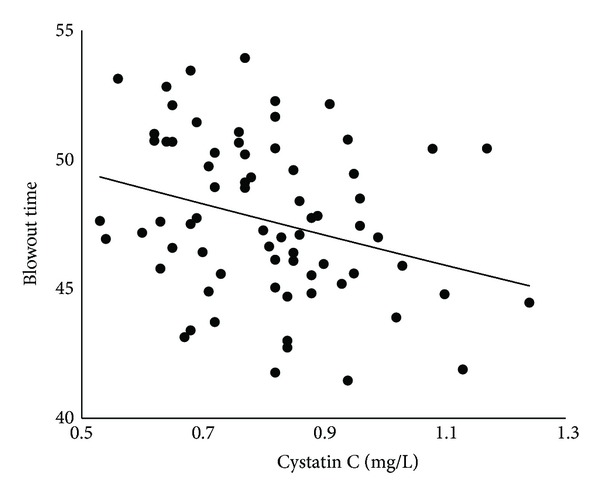
Correlation between blowout time and serum cystatin C in all subjects.

**Figure 5:**
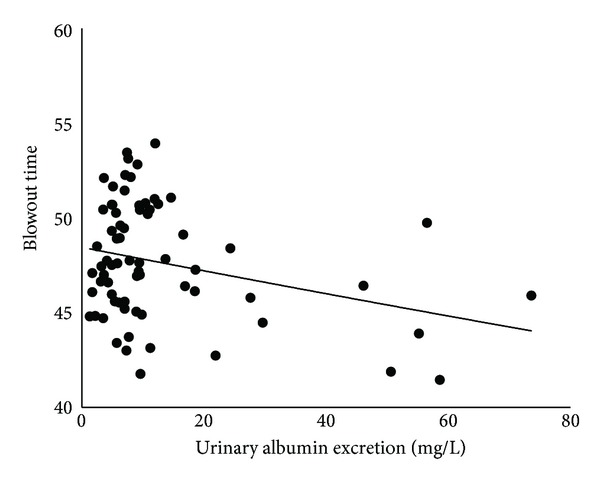
Correlation between blowout time and urinary albumin excretion in all subjects.

The findings of kidney function are given in Table 3. Twenty-two of the 71 (31.0%) patients had CKD. Pearson's coefficient of correlations between BOT and each of the systemic parameters is shown in Table 4. BOT was significantly correlated with age (*r* = −0.53, *t*  value = −5.13, *P* < 0.0001), SBP (*r* = −0.34, *t*  value = −3.00, *P* = 0.004), pulse pressure (*r* = −0.44, *t*  value = −4.05, *P* = 0.001), and heart rate (*r* = .40, *t*  value = 3.66, *P* = 0.0005). The IOP and OPP were not significantly correlated with BOT. All of the kidney function parameters were significantly correlated with BOT: creatinine, *r* = −0.24, *t*  value = −2.06, *P* = 0.04; estimated GFR, *r* = 0.42, *t*  value = 3.79, *P* = 0.0003; cystatin C, *r* = −0.29, *t*  value = −2.55, *P* = 0.01; and urinary albumin excretion, *r* = −0.29, *t*  value = −2.52, *P* = 0.01. The relationship between BOT and creatinine is shown in Figure 2, the estimated GFR in Figure 3, cystatin C in Figure 4, and that of urinary albumin excretion in Figure 5. All parameters in the CKD and without CKD cases were shown in Table 5. Age (70.4 ± 6.1 versus 58.6 ± 10.0 years, *P* < 0.0001) and incidence of hypertension (82 versus 45%, *P* = 0.008), creatinine (1.06 ± 0.17 versus 0.77 ± 0.14 mg/dL, *P* < 0.0001), and cystatin C (0.96 ± 0.12 versus 0.74 ± 0.11 mg/dL, *P* < 0.0001) were significantly higher in the CKD cases than in the without CKD cases. There was no significant difference in urinary albumin excretion. The BOT in the subjects with CKD cases was significantly lower than that in subjects without CKD cases (46.1 ± 3.0 versus 48.5 ± 2.9, *P* = 0.002).

## 4. Discussion

The increased risk of cardiovascular disease in patients with CKD is probably related to increased arterial stiffness measured by the pulse-wave velocity [16–21]. In addition, it was reported that a reduction of the estimated GFR may be associated with the presence and extent of abdominal aortic calcification [22]. Thus, there is evidence that kidney dysfunction such as CKD is associated with arteriosclerosis.

In an earlier study, we found that the BOT in the optic nerve head by LSFG was significantly correlated with age, bronchial pulse-wave velocity, intima-media thickness, and left ventricular diastolic function [14, 15]. These findings suggested that the changing MBR which reflects the ocular blood flow velocity becomes faster as the atherosclerotic changes worsen. Therefore, it was suggested that evaluations of the relationships between ocular circulation reflecting microcirculation and kidney function are very important for the general cardiac condition of the individual.

Nagaoka and Yoshida reported relationship between retinal blood flow, using Laser Doppler Velocimetry System, and chronic kidney disease in patients with type 2 diabetes mellitus [23]; however, to the best of our knowledge, the relationship between kidney function and ocular circulation using LSFG method has not been reported. The serum levels of creatinine, cystatin C, and urinary albumin excretion which are factors significantly associated with cardiovascular disease were evaluated as indicators of kidney function. The estimated GFR was also determined to assess the kidney function.

Our results showed that all of the kidney function parameters were significantly correlated with BOT, and the estimated GFR had the highest correlation with BOT. From the above results, it was suggested that BOT in the optic nerve head by LSFG can detect early kidney dysfunction. In addition, estimated GFR may be reflecting the ocular circulation more sensitive than other kidney function parameters. It was reported that, in the study population, the prevalence rate of low GFR and CKD increased with age [24]. And the prevalence of CKD was higher among hypertension, as previously reported [25]. Our result also supports the notion that prevalence of CKD is higher in hypertension and aging. In addition, the BOT in the subjects with CKD was significantly lower than that in subjects without CKD (*P* = 0.002). These findings indicate that BOT in the optic nerve head by LSFG may detect organ damage such as CKD. However, it is unknown whether kidney function affects ocular circulation directly or is a surrogate marker of kidney function. Thus, a more detailed investigation is needed. In current study, the region of measurement was set for the entire optic nerve head area. This area was selected because it had the highest correlation with all of kidney function in preliminary investigations than the other regions.

BOT was also significantly correlated with age, SBP, pulse pressure, and heart rate but not with the IOP and OPP. It should be noted that these factors affect the results of BOT.

There are limitations to this study. First, this study was cross-sectional with a small number of subjects. It will be necessary to conduct a prospective study with a larger number of subjects with and without kidney dysfunction. Second, in this study, patients who already developed coronary artery disease or heart failure were excluded, in order to standardize patient background. Thus, further studies will be needed to investigate the association between BOT and serious arteriosclerosis-related diseases such as cardiovascular diseases in comparison with parameters of kidney function.

In conclusion, our results confirmed that measurements of BOT in the optic nerve head by LSFG can be a useful method to not only large vascular function, but also age-related organ damage such as CKD. In addition, ocular circulation by LSFG method may reflect the early stage of kidney dysfunction without severe atherosclerotic disease such as cardiovascular events.

## Figures and Tables

**Table 1:** Characteristics of subjects.

Men : women	48 : 23
Age (years)	62.3 ± 10.5
Systolic blood pressure (mmHg)	126.8 ± 16.6
Diastolic blood pressure (mmHg)	71.4 ± 9.9
Mean arterial blood pressure (mmHg)	89.9 ± 10.9
Pulse pressure (mmHg)	55.4 ± 13.2
Heart rate (beat per minutes)	66.6 ± 10.8
Intraocular pressure (mmHg)	13.2 ± 2.2
Ocular perfusion pressure (mmHg)	46.7 ± 7.5
Body mass index (kg/m^2^)	24.0 ± 3.2
Hypertension (%)	40/71 (56.3)

Mean ± standard deviation, *n* = 71.

**Table 2:** Results of blowout time using laser speckle flowgraphy.

	Mean ± standard deviation	Range
Blowout time	47.8 ± 3.1	41.5 to 53.9

Mean ± standard deviation, *n* = 71.

**Table 3:** The results of serum creatinine, estimated glomerular filtration rate, serum cystatin C, and urinary albumin excretion as kidney function.

Creatinine (mg/dL)	0.86 ± 0.20
Estimated GFR (mL/min/1.73 m^2^)	66.9 ± 12.7
Cystatin C (mg/L)	0.81 ± 0.15
Urinary albumin excretion (mg/L)	12.8 ± 14.8

Presence of chronic kidney disease (%)	22/71 (31.0)

Mean ± standard deviation, *n* = 71.

GFR: glomerular filtration rate.

**Table 4:** Results of Pearson's correlation analysis between blowout time and all parameters.

Explanatory variables	*r*	*t* value	*P* value
Age (years)	−0.53	−5.13	<0.0001
Systolic blood pressure (mmHg)	−0.34	−3.00	0.004
Diastolic blood pressure (mmHg)	0.02	0.13	0.90
Mean arterial blood pressure (mmHg)	−0.16	−1.38	0.17
Pulse pressure (mmHg)	−0.44	−4.05	0.001
Heart rate (beat per minutes)	0.40	3.66	0.0005
Intraocular pressure (mmHg)	−0.04	−0.35	0.73
Ocular perfusion pressure (mmHg)	−0.15	−1.22	0.23
Body mass index (kg/m^2^)	0.08	0.67	0.50
Creatinine (mg/dL)	−0.24	−2.06	0.04
Estimated GFR (mL/min/1.73 m^2^)	0.42	3.79	0.0003
Cystatin C (mg/L)	−0.29	−2.55	0.01
Urinary albumin excretion (mg/L)	−0.29	−2.52	0.01

Objective variable: blowout time, *n* = 71.

eGFR: estimated glomerular filtration rate.

**Table 5:** Results of all parameters in patients with the chronic kidney disease (CKD) and without CKD.

	CKD (+)	CKD (−)	*P* value
Age (years)	70.4 ± 6.1	58.6 ± 10.0	<0.0001*
Men : women	18 : 4	30 : 19	0.15^†^
Systolic blood pressure (mmHg)	131.2 ± 19.0	124.9 ± 15.3	0.14*
Diastolic blood pressure (mmHg)	72.3 ± 11.4	71.0 ± 9.3	0.61*
Mean arterial blood pressure (mmHg)	91.9 ± 12.3	89.0 ± 10.2	0.29*
Pulse pressure (mmHg)	58.9 ± 15.6	53.9 ± 11.9	0.14*
Heart rate (beat per minutes)	65.1 ± 12.2	67.3 ± 10.2	0.44*
Intraocular pressure (mmHg)	13.2 ± 2.3	13.2 ± 2.2	0.94*
Ocular perfusion pressure (mmHg)	48.1 ± 8.5	46.1 ± 7.1	0.32*
Body mass index (kg/m^2^)	24.1 ± 2.6	24.0 ± 3.5	0.96*
Hypertension (%)	18/22 (82)	22/49 (45)	0.008^†^
Creatinine (mg/dL)	1.06 ± 0.17	0.77 ± 0.14	<0.0001*
Cystatin C (mg/L)	0.96 ± 0.12	0.74 ± 0.11	<0.0001*
Urinary albumin excretion (mg/L)	15.59 ± 17.31	11.61 ± 13.56	0.45^‡^
Blowout time	46.1 ± 3.0	48.5 ± 2.9	0.002*

Mean ± standard deviation, *n* = 71.

*Unpaired *t*-test.

^†^Yates 2 × 2 chi-square test.

^‡^Mann-Whitney *U* test.
